# High cytotoxicity and anti-proliferative activity of algae extracts on an in vitro model of human hepatocellular carcinoma

**DOI:** 10.1186/s40064-016-2938-2

**Published:** 2016-08-12

**Authors:** Celso Alves, Susete Pinteus, André Horta, Rui Pedrosa

**Affiliations:** MARE – Marine and Environmental Sciences Centre, ESTM, Polytechnic Institute of Leiria, 2520-641 Peniche, Portugal

**Keywords:** Bioactive compounds, Marine natural products, HepG-2 cells, Seaweeds, Antitumor activity, Cancer

## Abstract

**Background:**

Cancer represents a serious threat for human health with high social and economic impacts worldwide. Therefore, the development of new anticancer drugs is of most importance. The aim of the present study was to evaluate the antitumor potential of twelve algae from Portugal coast on an in vitro model of human hepatocellular carcinoma (HepG-2 cells).

**Results:**

Both extracts of *Asparagopsis armata* (1000 µg/ml; 24 h) presented high cytotoxicity with 11.22 ± 2.98 and 1.51 ± 0.38 % of HepG-2 live cells, respectively. *Sphaerococcus coronopifolius* methanolic and dichloromethane extracts (1000 µg/ml) also generated high reduction on HepG-2 viability (14.04 ± 2.62 and 12.84 ± 3.82 % of HepG-2 live cells, respectively). The most potent anti-proliferative activity was induced by dichloromethane extract (1000 µg/ml; 24 h) of *Sphaerococcus coronopifolius*, *Asparagopsis armata* and *Plocamium cartilagineum* with 99.61 ± 0.27, 98.56 ± 0.81 and 85.13 ± 1.04 % of cell’s proliferation reduction, respectively. *Sphaerococcus coronopifolius* dichloromethane extract exhibited the highest potency both on cytotoxicity and anti-proliferation assays with an IC_50_ of 14.1 and 32.3 μg/ml, respectively.

**Conclusions:**

*Sphaerococcus coronopifolius* is a promising source of new molecules with possible application on cancer therapeutics.

## Background

Nowadays, the malignant tumours are one of the major cause of death in humans and have high impact in industrialized countries (Fukahori et al. 2008; Smyrniotopoulos et al. 2010). In the last decades, the search and development of new drugs have increased, and nature became a relevant resource for the discovery of anticancer compounds. Today, more than 60 % of the commercially available anticancer drugs are of naturally origin. Naturally derived anti-proliferative drugs such as doxorubicin, bleomycin, daunomicin, vincristine, mytomicin C and vinblastine play an important role in curative cancer chemotherapy in a number of solid tumours and haematological malignancies (Jimeno et al. 2004; Sithranga Boopathy and Kathiresan 2010).

Marine organisms have already proved to be a rich source of bioactive compounds, and as a result, their exploration for pharmacological purposes have increased greatly along last years. Marine organisms survive and live within complex communities and also in close association with other organisms, consequently, they produce bioactive molecules normally as secondary metabolites in response to ecological pressures such as competition for space, maintenance of unfouled surfaces, deterrence of predation and the ability to successfully reproduce (Chin et al. 2006; Li et al. 2013; Salvador et al. 2007; Shanmughapriya et al. 2008). These compounds have evolved over millions of years as defence strategies against predators, to keep competitors at bay or to paralyze their prey (Haefner 2003). The marine environment represents a vast proportion of the earth’s biodiversity and it has been estimate that biological diversity in marine ecosystems may be higher than in tropical rain forests (Allsopp et al. 2009). This offers an abundance of highly potent secondary metabolites for exploitation as bioactive compounds. Moreover, it is widely accepted that marine natural products provide unusual and unique chemical structures upon which molecular modelling and chemical synthesis of new drugs can be based with greater efficacy and specificity for the treatment of many human diseases (Ebada et al. 2010; Jha and Zi-rong 2004; Stonik 2009). To date, many unique chemically compounds of marine origin with various biological activities have been isolated, and some of them are under investigation and are being used to develop new pharmaceuticals (Kijjoa and Sawangwong 2004). Among the marine organisms, algae have arisen as an emerging interest in the biomedical area and recent trends in macro-algae natural drug research are revealing the biomedical potential of these organisms in human diseases treatment. They are producers of bioactive substances, which show different biological activities, such as antioxidant, antimicrobial, antiviral, anthelmintic, anti-inflammatory, anticoagulant, antituberculosis, antiviral and antitumor (Chew et al. 2008; Mayer et al. 2009; Murray et al. 2013; Pinteus et al. 2015; Ye et al. 2008). Moreover, numerous algae and their metabolites have shown potent cytotoxic activities and various authors have suggested the consumption of algae as a chemopreventive measure against several cancers (Taskin et al. 2010; Yuan and Walsh 2006). Different groups of bioactive molecules with antitumor activity have been isolated from algae, such as sulfated polysaccharides, phenolic compounds, carotenoids and terpenoids. Such compounds have showed anti-proliferative activity in human cancer cell lines in vitro, as well as inhibitive activity in tumours growing in animal models (Kuo et al. 2011; Kwon et al. 2007; Lins et al. 2009; Thoppil and Bishayee 2011; Zandi et al. 2010). Therefore the aim of this study was to evaluate the antitumor potential of twelve algae from Peniche coast (Portugal) on an in vitro carcinoma model of human hepatocellular cancer (HepG-2 cells).

## Methods

### Sampling and identification of algae extracts

Algae were collected along the Peniche coast (Portugal), during June and July (2011), and immediately transported to the laboratory. They were cleaned and washed with sea water and then in fresh water to remove epiphytes, detritus and encrusting material. Algae were identified as *Asparagopsis armata*, *Ceramium ciliatum*, *Plocamium cartilagineum* and *Sphaerococcus coronopifolius* (Rhodophyta division); *Fucus spiralis*, *Halopteris filicina*, *Saccorhiza polyschides* and *Stypocaulon scoparium* (Heterokontophyta division); *Codium adhaerens*, *Codium tomentosum*, *Codium vermilara* and *Ulva compressa* (Chlorophyta division). Identification was performed by Dr. Susete Pinteus, supported by Marine Algae: Biodiversity, Taxonomy, Environmental Assessment, and Biotechnology guide (Pereira and Neto 2014). Finally algae were kept at −80 °C (Thermo, Electron Corporation) until extraction process.

### Preparation of algae extracts

Algae were previously frozen at −80 °C and then ground with a mixer grinder to make a powder. Each alga sample was sequentially extracted in 1:4 biomass:solvent ratio with methanol (>99 %, Fisher Scientific, United Kingdom) and dichloromethane (Fisher Scientific, United Kingdom) solvents at constant stirring for 12 h. Liquid–liquid extraction was also performed for the methanolic extract, using *n*-Hexane (Lab-Scan analytical Sciences, Poland). The solvents were evaporated in a rotary evaporator (Laborota 4000, Heidolph) at 40 °C and the extracts were then solubilized in dimethyl sulfoxide (Sigma, Germany) and stored at −20 °C until further use.

### Maintenance of cell culture

Human hepatocellular cancer model (HepG-2 cells) (ATCC HB-8065) was acquired from American Type Culture Collection (ATCC). HepG-2 cells were cultured in RPMI medium (Sigma, USA) supplemented with 10 % of fetal bovine serum (FBS) (Gibco, USA) and 1 % of antimycotic (100 U/ml penicillin G, 0.25 µg/ml amphotericin B and 100 µg/ml streptomycin) (Sigma, USA). Cell medium was changed every 3 days, and the cells reached confluence after 5–6 days of initial seeding. For subculture, cells were dissociated with tripsin-EDTA (Sigma, USA), split 1:3 and subculture in Petri dishes with 25 cm^2^ growth area. Cells were maintained in 95 % of humidified atmosphere, 5 % of CO_2_ and 37 °C of temperature.

### In vitro evaluation of cytotoxic and anti-proliferative activities

Algae activities were evaluated by decreases on cell viability and cell proliferation studies induced by methanolic and dichloromethane extracts. Cell viability studies were performed after cells reached the total confluence in 96-well plates. The algae extracts were dissolved in culture medium without FBS and sterile filtered (0.2 µm). Then, the extracts (1000 µg/ml) were incubated with cells during 24 h. For algae extracts with high effect after 24 h of incubation, the time-course effect was also evaluated after 12 h of incubation. For cellular proliferation studies, cells were seeded in 96-well plates and after 36 h of initial seeding, they were incubated during 24 h with algae extracts (1000 µg/ml), previously dissolved in cultured medium with FBS and sterile filtered (0.2 µm, Whatman, UK). The extracts which produced the most potent effects after 24 h incubation, the time-course effect was also evaluated (12 and 48 h).

Dose–response assays (10–1000 μg/ml; 24 h) were accessed for the extracts that exhibited the highest effects, both on the cytotoxicity and anti-proliferation assays. Cisplatin (Sigma, USA) and tamoxifen (Sigma, China) were used as standard drugs.

### HepG-2 cells viability and proliferation: 3-[4,5-dimethylthiazol-2-yl]-2,5-diphenyl tetrazolium bromide (MTT) colorimetric method

The antitumor capacity of algae extracts on cell viability and cell proliferation studies were measured using MTT method. This assay is based on cleavage of the tetrazolium salt MTT by mitochondrial dehydrogenases of viable cells to form a blue formazan product. The formazan produced is proportional to the number of living cells present and can be measured colorimetrically (Yuan and Walsh 2006). After the treatment with the methanol and dichloromethane extracts, the cells medium was removed and cells were washed with Hanks’ medium (medium composition in mM: NaCl 137, KCl 5, MgSO4 0.8, Na2HPO4 0.33, KH2PO4 0.44, CaCl2 0.25; MgCl2 1.0, Tris HCl 0.15 and sodium butyrate 1.0, pH 7.4). After that, cells were incubated with MTT (1.2 mM) (Sigma, Germany), previously dissolved in Hanks’ medium, during 4 h at 37 °C. The formazan products were dissolved in isopropanol (Panreac, Spain) with 0.04 N HCl and the absorbance (Abs) was read at 570 nm (Biotec, Synergy H1 hybrid reader).

### HepG-2 cells viability and proliferation: calcein-AM fluorescent method

The calcein-AM method is a fluorescent method which evaluates the cell esterases activity in order to estimate the cell viability and proliferation. The cell membranes are permeable to calcein-AM, a non-fluorescent dye, which is taken up and converted by intracellular esterases to calcein. The calcein remains trapped within the cell emitting green fluorescence that can measured (Pedrosa and Soares-da-Silva 2002).

In order to confirm the results obtained before, extracts that exhibit high activity in the MTT assay were also tested by calcein-AM method. After treatment with algae extracts, cells were washed twice times with Hanks’ medium and loaded with 2 µM of calcein-AM (Invitrogen, USA), previously dissolved in Hanks’ medium, at room temperature for 30 min. The natural fluorescence of the cells was determined on wells incubated with Hanks medium without calcein-AM. Fluorescence (F) was measured at 485 nm excitation and 530 nm emission wavelengths in a multiplate reader (Biotec, Synergy H1 hybrid reader).

The results obtained for both studies were expressed in percentage of control:

$$(\% \,{\text{of}}\,{\text{control}}) = ({\text{Abs}}\,{\text{or}}\,{\text{F}}\,{\text{sample}}/{\text{Abs}}\,{\text{or}}\,{\text{F}}\,{\text{control}}) \times 100.$$

### Statistical analysis

One-way analysis of variance (ANOVA) was used followed by Dunnett test to discriminate significant differences between extracts and controls or vehicle. These analyzes were performed using GraphPad InStat for Windows. Results are presented as mean ± standard error of mean (SEM). The significance level was inferred at p < 0.05 or p < 0.01 for all statistical tests. The IC_50_ concentration was calculated from nonlinear regression analysis using the GraphPad Prism software with the equation: ${\text{Y}} = 100/[1 + 10^{{({\text{X}} - {\text{LogIC}}_{50} )}} ]$.

## Results

### Cytotoxicity activities of algae extracts: HepG-2 cell viability

**Fig. 1:**
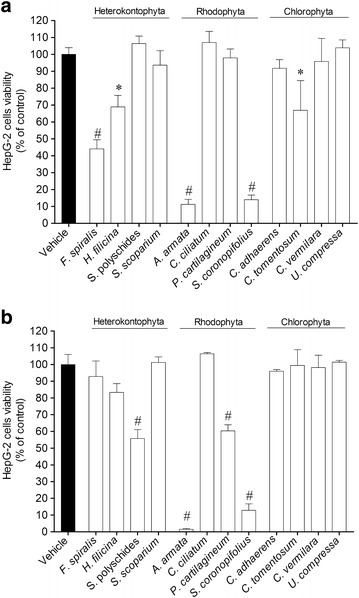
The effects on HepG-2 cells viability (% of control) induced by methanolic (**a**) and dichloromethane (**b**) extracts (1000 µg/ml) after 24 h of incubation—MTT method. *Each column* represents the mean of 8 experiments per group; *vertical lines* show SEM. ^#^p < 0.01 and *p < 0.05 compared with control

The methanolic and dichloromethane extracts obtained from twelve algae were tested for their ability to induce cytotoxicity on HepG-2 cells (1000 µg/ml; 24 h) (Fig. 1). In the methanolic extract the major potential was exhibited by algae that belong to Rhodophyta and Heterokontophyta division. The methanolic extracts of red algae *Asparagopsis armata* (11.22 ± 2.98 % of HepG-2 live cells) and *Sphaerococcus coronopifolius* (14.04 ± 2.62 % of HepG-2 live cells) showed high cytotoxicity on HepG-2 cells, inducing a cell viability decrease in more than 80 % (Fig. 1a). As regards to dichloromethane extracts, the highest activity was achieved by *A. armata* and *S. coronopifolius* extracts which exhibited capacity to reduce cell viability in more than 80 % (Fig. 1b). Algae belonging to Chlorophyta division did not showed significant cytotoxicity activity on HepG-2 cells (Fig. 1).

**Fig. 2:**
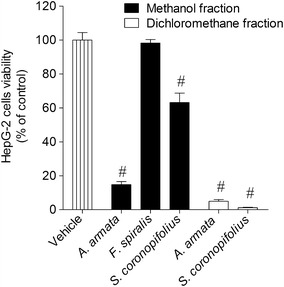
The effects on HepG-2 cells viability (% of control) induced by methanolic and dichloromethane extracts (1000 µg/ml) after 24 h of incubation—calcein-AM method. *Each column* represents the mean of 8 experiments per group; *vertical lines* show SEM. ^#^p < 0.01 compared with control

**Fig. 3:**
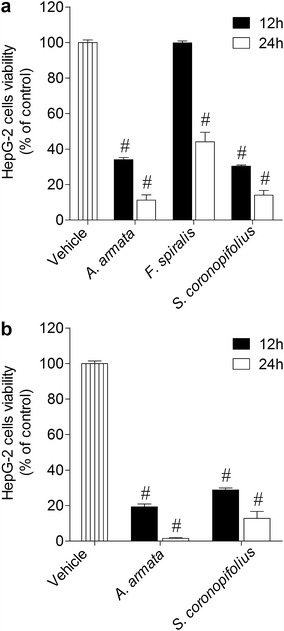
The effects on HepG-2 cells viability (% of control) induced by methanolic (**a**) and dichloromethane (**b**) extracts (1000 µg/ml) after 12 and 24 h of incubation—MTT method. *Each column* represents the mean of 8 experiments per group; *vertical lines* show SEM. ^#^p < 0.01 compared with control

The extracts that showed capacity to reduce cell´s viability in more than 50 % were also tested through the calcein-AM fluorescent method. Regarding to the methanolic extract, *A. armata* presented similar results to those obtained in the MTT method, however *S. coronopifolius* and *F. spiralis* presented much less activity in the reduction of cell viability. On the other hand, the dichloromethane extracts showed similar activity in the reduction of the cell viability in both methods; however *S. coronopifolius* presented an even more marked effect in the calcein-AM method (Fig. 2). Dose–response assays were accessed in order to define the potency of the effects. As it is shown on Table 1, the dichloromethane extract of *S. coronopifolius* exhibited the lowest IC_50_ (14.1 µg/ml). For the standard drugs, only cisplatin exhibited cytotoxicity on HepG-2 cell (IC_50_: 136.5 µg/ml) (Table 1). The time-course effect was also evaluated for the extracts that displayed the highest decrease (1000 µg/ml; 24 h) on the HepG-2 cells viability. In this assay cells were incubated during 12 and 24 h with extracts at 1000 µg/ml. For both extracts, a time-dependent effect was observed. However, during the first 12 h of incubation, the effect induced by *S. coronopifolius* and *A. armata* methanol extracts were more marked when compared with the *F. spiralis* methanolic extract-induced HepG-2 cell death (Fig. 3).

**Table 1:** IC_50_ values obtained for methanolic and dichloromethane extracts (µg extract/ml) that presented the highest cytotoxicity activity on HepG-2 cells

Algae	Extract	
	Methanol IC_50_ (µg/ml)	Dichloromethane IC_50_ (µg/ml)
*Fucus spiralis*	739.4 (521.9–1047.0)	–
*Asparagopsis armata*	567.9 (317.7–1015.0)	473.1 (341.9–654.7)
*Sphaerococcus coronopifolius*	470.6 (310.7–712.6)	14.1 (8.1–24.6)

Values are expressed as means with 95 % confidence intervals. Cisplatin and tamoxifen were used as standard drugs. Cisplatin exhibited an IC_50_ of 136.5 µg/ml (116.8–159.5) and tamoxifen did not showed cytotoxicity

### Anti-proliferative activity of algae extracts: HepG-2 cells proliferation

**Fig. 4:**
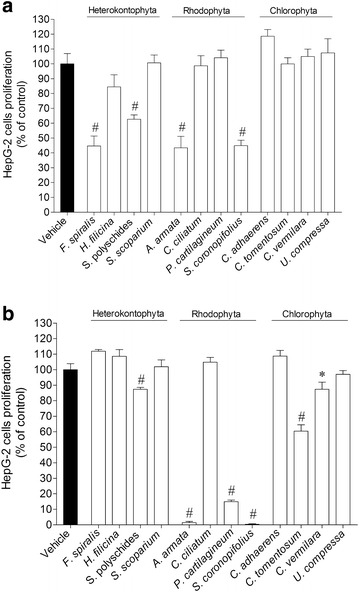
The effects on HepG-2 cells proliferation (% of control) induced by methanolic (**a**) and dichloromethane (**b**) extracts (1000 µg/ml) after 24 h of incubation—MTT method. *Each column* represents the mean of 8 experiments per group; *vertical lines* show SEM. ^#^p < 0.01 and *p < 0.05 compared with control

To test the effect of the methanol and dichloromethane extracts on HepG-2 cells proliferation, cells were incubated with extracts during 24 h at 1000 µg/ml. As shown in Fig. 4a, the methanolic extracts of algae belonging to Chlorophyta group did not show any activity on HepG-2 cells proliferation. On the other hand, *F. spiralis* (44.60 ± 6.75 % of HepG-2 cell proliferation), *S. polyschides* (62.66 ± 2.95 % of HepG-2 cell proliferation), *A. armata* (43.42 ± 7.69 % of HepG-2 cell proliferation) and *S. coronopifolius* (44.87 ± 3.64 % of HepG-2 cell proliferation) showed the highest inhibitory effects on the cell proliferation. However, the highest inhibition was induced by dichloromethane extract of *A. armata* (1.44 ± 0.81 % of HepG-2 cell proliferation), *P. cartilagineum* (14.87 ± 1.04 % of HepG-2 cell proliferation) and *S. coronopifolius* (0.39 ± 0.27 % of HepG-2 cell proliferation), that inhibited the cellular proliferation of HepG-2 cells in more than 80 % (1000 µg/ml; 24 h) (Fig. 4b).

**Fig. 5:**
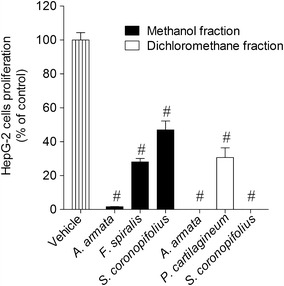
Effects on HepG-2 cells proliferation (% of control) induced by methanolic and dichloromethane extracts (1000 µg/ml) after 24 h of incubation—calcein-AM method. *Each column* represents the mean of 8 experiments per group; *vertical lines* show SEM. ^#^p < 0.01 compared with control

The extracts that revealed capacity to inhibit the HepG-2 cell’s proliferation in more than 50 % were also tested through the fluorescent method as in cell viability study. As demonstrated in Fig. 5, all the selected extracts had inhibitory effect on HepG-cellular proliferation as it was previously verified by the MTT method. The potency of the effects was evaluated by testing different concentrations of extract (10–1000 µg/ml) on HepG-2 cells proliferation with an incubation period of 24 h. The lowest IC_50_ was exhibited by the dichloromethane extract of *S. coronopifolius* (32.3 µg/ml). For the standard drugs, the IC_50_ exhibited by cisplatin and tamoxifen was 22.63 µg/ml and 16.97 µg/ml, respectively (Table 2).

**Table 2:** IC_50_ values obtained for methanolic and dichloromethane extracts (µg extract/ml) that presented the highest anti-proliferative activity on HepG-2 cells

Algae	Extract	
	Methanol IC_50_ (µg/ml)	Dichloromethane IC_50_ (µg/ml)
*Fucus spiralis*	1039.0 (603.7–1789.0)	–
*Asparagopsis armata*	857.3 (524.5–1401.0)	518.9 (341.3–789.0)
*Plocamium cartilagineum*	–	852.7 (471.9–1541.0)
*Sphaerococcus coronopifolius*	646.5 (398.4–1049.0)	32.3 (22.4–46.7)

Values are expressed as means with 95 % confidence intervals. Cisplatin and tamoxifen were used as standard drugs. Cisplatin and tamoxifen exhibited an IC_50_ of 22.63 µg/ml (18.24–28.07) and 16.97 µg/ml (11.83–24.35), respectively

**Fig. 6:**
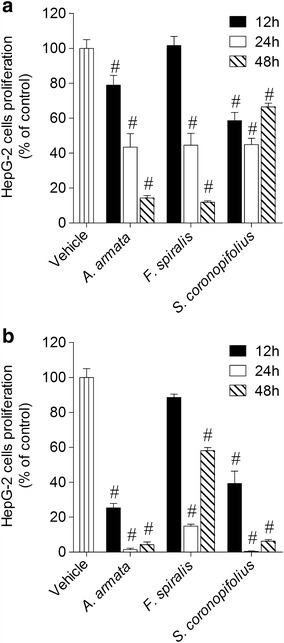
Effects on HepG-2 cells proliferation (% of control) induced by methanolic (**a**) and dichloromethane (**b**) extracts (1000 µg/ml) after 12, 24 and 48 h of incubation—MTT method. *Each column* represents the mean of 8 experiments per group; *vertical lines* show SEM. ^#^p < 0.01 compared with control

The time-course effect was evaluated by testing the algae extracts (1000 µg/ml) on HepG-2 cells proliferation at 12, 24 and 48 h (Fig. 6). In both extracts, all algae extracts showed a time dependent effect between 12 and 24 h. However, only the methanolic extracts of *A. armata* and *F. spiralis* exhibited a time dependent inhibition of cell proliferation after 48 h of incubation.

## Discussion

Algae have caused an emerging interest in the biomedical area, mainly due to their content of biodynamic compounds with therapeutic value. These compounds are providing valuable ideas for the development of new drugs against different diseases (Ayesha et al. 2010; Ye et al. 2008). In this study, the antitumor potential of different algae from Peniche coast was evaluated on an in vitro model of human hepatocellular carcinoma (HepG-2 cells). Among all the tested algae, *Asparagopsis armata*, *Fucus spiralis*, *Plocamium cartilagineum* and *Sphaerococcus coronopifolius* presented the highest potential in both cytotoxicity and anti-proliferative studies. The activity evidenced by these four algae can be associated with the presence of sulfated polysaccharides, since in recent years these molecules have been reported with anti-proliferative activity on in vitro cancer models (cancer cellular lines), as well as inhibitive activity in tumours growing in mice. In addition, they have anti-metastatic activity blocking the interactions between cancer cells and the basement membrane (Costa et al. 2010; Rocha et al. 2005). Sulphated polysaccharides are found in varying amounts in three major divisions of marine algae, Rhodophyta (red algae), Heterokontophyta (brown algae) and Chlorophyta (green algae) (Wijesekara et al. 2011). The activity exhibited by brown algae, namely by *Fucus spiralis* can be related with the presence of fucoidans, which are sulfated polysaccharides produced only by brown algae. These molecules have been isolated from different species belonging to *Fucus* genus and have showed capacity to inhibit the growth, the angiogenesis and the invasion progressions of tumor cells (Ale et al. 2011; Hyun et al. 2009; Imbs et al. 2009; Kamihira et al. 2010; Skriptsova et al. 2012; Ye et al. 2005). Other group of sulphated polysaccharides, the heterofucans, were isolated and showed strong anti-proliferative activity against HeLa cells and moderate activity on HepG-2 cells (Costa et al. 2011). A similar effect was observed with diterpenes isolated from the brown algae *Dictoyota dichotoma*, which exhibited a potent cytotoxicity on HepG-2 cells (Ayyad et al. 2011). Actually, terpenoids and polysaccharides are strong candidates to mediate the cytotoxic effect associated with alga *Fucus spiralis*. This fact is reinforced since these molecules can be extracted with methanol (polar), precisely the extract that revealed the highest activity in this study.

Red algae also showed interesting results on HepG-2 cells. Among these, *A. armata*, *P. cartilagineum* and *S. coronopifolius* had the highest effects on HepG-2 cells. Several studies have reported the antimicrobial activity of *A. armata* against different types of microorganisms, showing that this alga produce compounds with pharmacological potential (Genovese et al. 2009; Paul et al. 2006; Zbakh et al. 2012); however the cytotoxicity effects of this algae in cells lines were only reported by Zubia et al. (2009). On the other hand, the activity demonstrated by *P. cartilagineum* may be due to the presence of halogenated monoterpenes, since these molecules were already isolated from this alga and revealed high activity on SW480 cells (Inés et al. 2004). Nevertheless, the best activity in both studies was exhibited by the dichloromethane extract of *S. coronopifolius* that exhibited the lowest IC_50_ on cell viability and cell proliferation studies. These potential is not surprising since previous studies isolated diterpenes with great interest from *S. coronopifolius* collected in Mediterranean Sea (Piazza et al. 2011; Smyrniotopoulos et al. 2009). These molecules showed interesting results against several cells lines and were able to overcome the natural resistance of certain tumour cells to apoptosis (Smyrniotopoulos et al. 2008, 2010).

The potential of *S. coronopifolius* gets even more interesting when compared with the standard drugs, cisplatin and tamoxifen. In the anti-proliferative tests, the IC_50_ exhibited by the *S. coronopifolius* dichloromethane extract was similar to the IC_50_ exhibited by these antitumor drugs. By other side, in the cytotoxicity test, the dichloromethane extract of *S. coronopifolius* displayed an IC_50_ smaller than those drugs.

On the other hand, the environmental conditions to which algae are subjected can result in the production of different molecules, and the possibility of the presence of new molecules involved in the cytotoxicity and anti-proliferative effects presented in this work must be considered. Moreover, several authors have associated the production of compounds with toxicity and anti-proliferative activities to temporal-space variations, depending on the community or the season, pointing to the important role of biotic and abiotic factors (Marti et al. 2004; Osman et al. 2010; Taskin et al. 2010). These factors can also explain the absence of positive results of the algae belonging to Chlorophyta division. These did not show any potent activity on HepG-2 cells; however, previous studies reported anti-proliferative activity for green algae (Chlorophyta) on different cells lines (Costa et al. 2010; Kim et al. 2013; Villarreal-Gómez et al. 2010).

These results indicate the possible presence of compounds with huge potency. Moreover, the extracts here tested are a complex mixture of compounds and the portion of active compounds may be very low. These data support the view that *S. coronopifolius* can be an interesting source of molecules with antitumor potential.

## Conclusions

In summary, the screening of this study allowed to select four algae from twelve with interesting antitumor potential on HepG-2 cells, namely *Fucus spiralis*, *Asparagopsis armata*, *Plocamium cartilagineum* and *Sphaerococcus coronopifolius*, being the highest activity exhibited by *S. coronopifolius*. Although this algae can be a promising source of new molecules with antitumor activity on hepatocellular carcinoma, this study is one of many steps required in order to obtain a therapeutic molecule. Further on it will be required to isolate, identify and purify the molecules with antitumor potential and also evaluate the intracellular signal pathways linked to the cell death mechanism or/and to the cell cycle regulation. Nevertheless, for our knowledge, this is the first antitumor screening on algae from the Peniche coast revealing high antitumor potential that opens a new opportunity window for cancer therapeutics research.
